# Experiences regarding nutrition and exercise among women during early postpartum: a qualitative grounded theory study

**DOI:** 10.1186/s12884-019-2508-z

**Published:** 2019-10-21

**Authors:** Beth Murray-Davis, Lindsay Grenier, Stephanie A. Atkinson, Michelle F. Mottola, Olive Wahoush, Lehana Thabane, Feng Xie, Jennifer Vickers-Manzin, Caroline Moore, Eileen K. Hutton

**Affiliations:** 1 McMaster Midwifery Research Center, Department of Obstetrics and Gynecology, McMaster University, Hamilton, ON Canada; 2 Department of Pediatrics, McMaster University, Hamilton, ON Canada; 3 R. Samuel McLaughlin Foundation- Exercise and Pregnancy Lab, School of Kinesiology, The University of Western Ontario, London, ON Canada; 4 Global Health, McMaster University, Hamilton, ON Canada; 5 School of Nursing, McMaster University, Hamilton, ON Canada; 6 Department of Health Research Methods, Evidence and Impact, McMaster University, Hamilton, ON Canada; 7 Biostatistics Unit, Father Sean O’Sullivan Research Centre, St Joseph’s Healthcare Hamilton, Hamilton, ON Canada; 8 Public Health Services-Healthy Families, Healthy & Safe Communities, City of Hamilton, Hamilton, ON Canada

**Keywords:** Gestational weight gain, Postpartum weight retention, Postpartum women, Exercise, Nutrition, Weight loss, Body image, Guidelines for gestational weight gain

## Abstract

**Background:**

Excess gestational weight gain has long- and short-term implications for women and children, and postpartum weight retention is associated with an increased risk of long-term obesity. Despite the existence of dietary and exercise guidelines, many women struggle to return to pre-pregnancy weight. Experiences of women in tackling postpartum weight loss are poorly understood. We undertook this study to explore experiences related to nutrition, exercise and weight in the postpartum in women in Ontario, Canada.

**Methods:**

This was a nested qualitative study within The Be Healthy in Pregnancy Study, a randomized controlled trial. Women randomized to the control group were invited to participate. Semi-structured focus groups were conducted at 4–6 months postpartum. Focus groups were audio recorded, transcribed verbatim, coded and analyzed thematically using a constructivist grounded theory approach.

**Results:**

Women experienced a complex relationship with their body image, due to unrealistic expectations related to their postpartum body. Participants identified barriers and enablers to healthy habits during pregnancy and postpartum. Gestational weight gain guidelines were regarded as unhelpful and unrealistic. A lack of guidance and information about weight management, healthy eating, and exercise in the postpartum period was highlighted.

**Conclusion:**

Strategies for weight management that target the unique characteristics of the postpartum period have been neglected in research and in patient counselling. Postpartum women may begin preparing for their next pregnancy and support during this period could improve their health for subsequent pregnancies.

**Trial registration:**

NCT01689961 registered September 21, 2012.

## Background

Approximately half of all pregnant women in Canada gain in excess of the Institute of Medicine’s guidelines for gestational weight gain (GWG) [1–3]. Excess GWG occurs for up to three quarters of women who enter pregnancy overweight and for 40% of those of normal pre-pregnancy weight [4]. Excess GWG is associated with an increased risk of short- and long-term adverse outcomes. Women who gain above the GWG guidelines retain, on average, 3–4 kg up to 21 years postpartum compared to women who gain within the guidelines [5]. Approximately 25% of women have major postpartum weight retention, defined as > 4 kg, at 1 year postpartum [5–7] and 20% of women move into a higher body mass index (BMI) category at 18 months postpartum, regardless of pre-pregnancy BMI [5]. Weight retention between pregnancies places women at a greater risk for long-term obesity and future co-morbidities such as diabetes and cardiovascular disease [8]. Weight retention postpartum is a significant contributor to long-term obesity in women, suggesting that this transitional period may be a critical time for weight management and weight loss interventions for women’s health, and for the health of future pregnancies [6, 9, 10]. For the offspring, excess maternal GWG also poses short- and long- term risks including large for gestational age birth weight, hypoglycemia, hyperbilirubinemia [11–14] as well as higher risk during infancy, childhood and adolescence of diabetes mellitus, obesity, and hypertension [15–21].

Postpartum care typically includes monitoring the health of the mother within the first weeks following birth and concludes with a final assessment of health and wellbeing at 4–6 weeks postpartum [22, 23]. Women face unique challenges in managing their weight during this period, such as lack of time, motivation, social supports, and child-care [5]. It is difficult for new mothers to balance the demands of a newborn with other responsibilities such as children and work outside of the home [5]. Many postpartum women are unsuccessful in returning to their pre-pregnancy weight despite counselling from healthcare providers and the existence of numerous weight, dietary, and exercise guidelines [24–27]. However, counseling and guidelines are not likely to be successful unless the broader context of women’s knowledge, attitudes and psychosocial wellbeing are considered [28]. This is critical for identifying modifiable behavior that impacts weight gain and retention [29, 30]. The goal of the current research was to explore women’s experiences related to nutrition, exercise and weight gain postpartum.

## Methods

**Fig. 1:**
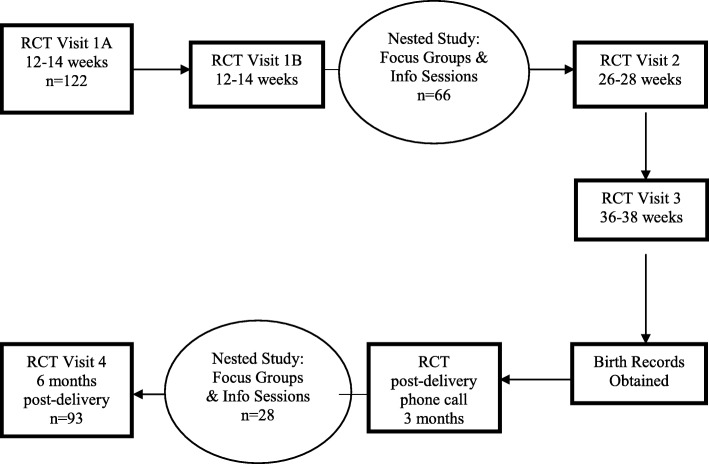
Study flow chart

This was a qualitative, constructivist grounded theory study nested within a randomized controlled trial (RCT; NCT01689961) examining the likelihood of attaining optimal GWG through an exercise and nutrition intervention [31]. The primary study protocol is described in detail elsewhere [31] and summarised here. Healthy pregnant women were recruited from two urban centres in Southwestern Ontario using posted advertisements at local hospitals and service organizations, as well as at medical and midwifery offices. All participants gave written informed consent and those who agreed to participate in the trial were randomized to either the intervention or the control group, receiving usual care. The latter included a single session at the beginning of the study (about 12–17 weeks gestation) with the study nutritionist who reviewed the Health Canada recommendations for nutrition and exercise during pregnancy [32] and provided the relevant written materials to the participants and to the health practitioner responsible for their pregnancy care. Members of the control group were invited to participate in the qualitative study. The nested qualitative study acted as a mechanism for retaining participants in the control group since evidence suggests that intervention studies looking at weight gain have high dropout rates, up to 41%, for studies citing ‘standard care’ as the control protocol [33–35]. To address this issue proactively, we developed a novel, yet benign, qualitative study addition to enhance engagement and retention of controls, with minimal risk of contamination, and which contributed to the creation of new knowledge [36]. All participants in the control group were invited to participate in focus groups and information sessions at 16–24 weeks gestation and again at 4–6 months postpartum (Fig. 1). The information sessions addressed pain relief options and breastfeeding for prenatal women and goal setting for health behaviours for postpartum women. The information sessions avoided topics directly related to the study to avoid contamination. Ethical approval was received from the Research Ethics Board of Hamilton Health Sciences and the Western University Health Sciences Research Ethics Board. Data were collected using semi-structured focus groups that were 60–90 min in duration. The focus groups took place between July 2014 and October 2016, when the participants were approximately 4–6 months postpartum. Focus groups were only run when a minimum of three participants could attend; if this requirement could not be met due to participant availability the focus groups were cancelled. Since our data collection was also a strategy for maintaining contact with the control group, we invited and encouraged all women to participate in the focus groups. Typically grounded theory data collection would cease once theoretical saturation was reached [37]. Saturation can usually be achieved within 2–6 focus groups, identifying 80–90% of themes [37]. Our data collection, however, continued until the RCT was complete to maximize participant retention [38, 39]. We performed an iterative review of our data which indicated that saturation was achieved, with no new data emerging in the last few focus groups [40].

Focus groups were facilitated by non-clinician research assistants, both female and male, who were experienced interviewers. Focus groups were audio recorded. The semi-structured question guide was developed to reflect typical issues that women consider during pregnancy and postpartum based on the literature [28, 41–51] and information addressed in prenatal classes [52, 53]. The interview guide was initially piloted with three women and then continually refined using theoretical sampling in a constant comparative process and as themes became saturated. This ensured the questions evolved during data collection to constantly build and refine the emerging theory (Table 1) [54–56]. Decisions to refine the focus group questions were carried out by the facilitator and Principal Investigator through interim peer debriefing meetings with the research team. Transcripts were sent to the participants for member-checking to ensure they were an accurate account of the focus group [57, 58].

**Table 1:** Focus group semi-structured question guide

General Questions about Pregnancy Experiences	
• How satisfied were you with how you managed your own care related to nutrition, exercise, and weight gain during your pregnancy? • What were some of the challenges or things that made it easy to get the information you wanted about your health in pregnancy? ◦ Probe: To what extent do you feel you could control these factors? • How did your nutrition and exercise practices change during pregnancy? ◦ Please describe any specific pregnancy issues that impacted this. • How realistic were the goals for weight gain set by you or your health care provider? • Please describe things you did to manage your weight gain in your pregnancy. • Please describe any pregnancy related complications you experienced associated with diet, exercise or weight gain. • How did your body image change during your pregnancy?	
General Questions about Postpartum Wellness	
• How do you feel about nutrition and exercise now that you have your baby? • What are some challenges or things that make it easy to enjoy healthy eating and exercise at this point in your life? • How concerned are you about weight loss in this postpartum period? • How easy do you think it will be to lose pregnancy weight? • What will be the main ways in which you plan to lose weight gained in pregnancy? • What type of help would be useful while trying to lose weight gained in pregnancy? • How would you approach eating, exercise and weight gain differently in a future pregnancy? • What has been an important lesson you have learned about wellness during this pregnancy and postpartum time period?	

The focus groups were transcribed verbatim and analyzed by two experienced, non-clinician research assistants using a constructivist grounded theory approach [59, 60]. Each transcript was read line-by-line and open-coded by two research assistants [54]. Axial coding using NVivo software (QSR International, Burlington, MA) allowed clustering of related codes into categories. These categories were grouped together to form themes through the process of selective coding. These themes were used to understand and describe the women’s experiences, beliefs and behaviours [55]. These themes came together to represent the emerging theory but remained grounded in the data from the women [39]. The Principal Investigator reviewed the coding at every stage of analysis to ensure rigour. Investigator triangulation was used to review, validate, challenge and come to a consensus on all codes, categories and themes by the two research assistants and Principal Investigator conducting the analysis. Interim analyses were shared with the larger research team at debriefing meetings during data analysis to confirm this process and ultimately reviewed and confirmed the audit trail. Theoretical saturation, theoretical sampling, peer debriefing meetings, member checking, audit trail and triangulation of data analysis strengthened the credibility, dependability and confirmability of the findings [57, 61–63].

The study team are experienced researchers from a variety of disciplines, bringing their unique perspectives from obstetrics, pediatrics, research methods, midwifery, public health, global health, nutrition and exercise in pregnancy. The research team had the explicit aim to help people achieve healthy behaviours in pregnancy and to determine what may be preventing that from happening. We approached data analysis with a healthcare lens, which involved experts in several health science fields and health clinicians: BMD is a Registered Midwife (RM) and OW is a Registered Nurse (RN). All authors except L.T. and F.X. are female. The research team entered the study holding the belief that women’s weight in pregnancy is an important topic and that excessive gestational weight gain results in adverse outcomes for both mother and offspring. The researchers who conducted and analyzed the focus groups were non-clinicians and had no data on BMI, weight gain or health data of any kind on the individuals who chose to participate.

## Results

**Fig. 2:**
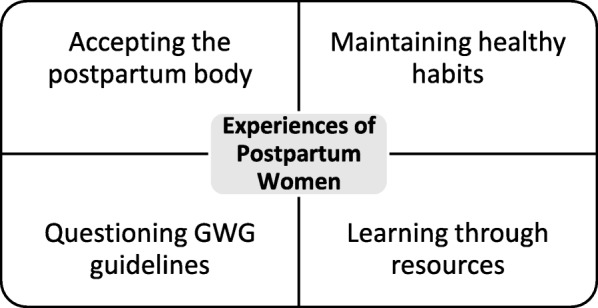
Themes informing postpartum women’s experiences

Baseline characteristics for the original 122 participants randomized to the control group were collected at 12–17 weeks gestation (Table 2). Of the 122 women randomized to the control group, 93 remained enrolled in the study until the postpartum time period (Fig. 2). We conducted eight focus groups with between three and six participants who were 4–6 months post-delivery, with a total of 28 women, 30.1% of all participants who completed the final study visit. Analyses of the data identified four major themes that emerged from the participants’ responses (Fig. 2).

**Table 2:** Baseline characteristics at 12–17 weeks gestation

Characteristics	Control group (*n* = 122)
Maternal age (y) mean ± SD	31.3 ± 4.3
University education n (%)	91 (74.6)
Pre-pregnancy BMI (kg/m^2^) mean ± SD	25.3 ± 4.6
Pre-pregnancy BMI (kg/m^2^) category n (%)	
Underweight (< 18.5)	2 (1.6)
Normal weight (18.5–24.9)	62 (50.8)
Overweight (25.0–29.9)	37 (30.3)
Obese (≥30)	21 (17.2)
Race/ethnicity n (%)	
European Descent	107 (87.7)
Mixed/Other	13 (10.7)
Unknown	2 (1.6)
Total family income n (%)	
< $45,000	10 (8.2)
$45,000–$74,999	25 (20.5)
> $75,000	78 (63.9)
Unknown	9 (7.4)
Married/living with significant other n (%)	114 (93.4)
Nulliparous n (%)	56 (45.9)

### Accepting the postpartum body

Participants’ perception of their body changed significantly from pre-pregnancy through to the postpartum period. The majority of participants described lowered self-image postpartum due to body changes, unrealistic expectations of postnatal recovery and ill-fitting clothing. This negativity was grounded in an external frame of reference where the women experienced, or perceived external judgement based on their physical appearance or behaviours. In the first trimester they felt worse about their bodies because they were gaining weight but there wasn’t a visible reason to outside observers. Once they started showing in the second trimester, they stated they felt better about their body since there was a ‘legitimate’ reason for their weight gain. After birth, however, they no longer had an ‘excuse’ for their larger body size as one person explained:

“*I love being pregnant. I feel like I can look good, like I can wear whatever I want and it’ll look good because I’ve got the belly and it doesn’t matter. Whereas when you’re not pregnant, you’re judged a lot more, body image is so much more…looked upon and judged but not if you’re pregnant.”*

Women described deriving satisfaction with their appearance from how they looked in clothing, whereas ill-fitting clothing negatively impacted their confidence and level of satisfaction with their body, as one woman described:

“*I struggle more now, I think because I feel like I don’t have anything to wear that looks nice. I don’t fit into my pre-pregnancy clothes and I’m too small for maternity clothes, and I don’t really want to wear them anyways, so I’m just in this weird in-between that makes me feel self-conscious and uncomfortable. But I think if I were to go out and buy a whole bunch of clothes that fit me properly I’d feel better, I just don’t want to stay here [at this weight] so I don’t want to spend the money.”*

Many stated they entered the postpartum period with a plan to eat well, exercise, lose weight to ‘get their body back’ and still juggle their regular responsibilities with caring for a newborn. One person reflected that her postpartum experience and body was far different than what she had expected: *“I think I struggled more after he came. Just, maybe having an idea in my head of wanting to lose the weight quicker than I have.”* Some women felt very eager to start exercising and lose weight but found that they had not given their bodies enough time to recover physically to feel comfortable and safe exercising. They described feelings of guilt about not regaining their fitness levels postpartum. The media portrayal of the postpartum body was another source of external pressure or perceived judgement which impacted body image negatively since women’s postpartum experience was very different from the portrayal of ‘bouncing back’ after baby:

“*You see the stories on the web about the underwear model who had a baby and two weeks later she’s back down to her pre-pregnancy size or less than her pre-pregnancy weight. You know, it’s not realistic. “*

Participants reported feeling disappointed by not achieving their postpartum weight and nutrition goals as quickly as they thought they should have. Many women realized their expectations were unrealistic, however, this didn’t lessen the impact it had on how they felt towards their bodies and weight loss journey.

Women’s views about their bodies were often unrelated to their feelings about their weight. For example, losing weight or reaching their pre-pregnancy weight would appear to be an accomplishment, however, many women articulated dissatisfaction with their bodies even after reaching this goal, *“I was back down to my pre-pregnancy weight within two weeks, but…as much as the number is the same…it’s not distributed the way it was before.”* Weight redistribution, loose skin, stretch marks and loss of muscle definition were consequences of pregnancy that also impacted body image.

Conversely, a minority of women, typically those who had more than one child described being happy with changes brought about by their pregnancies. Increased breast size or an increase in weight was a welcome change to some who had previously been self-conscious about their smaller size. Rather than being critical of themselves, some participants reported a shift toward viewing their body by its’ accomplishments, rather than its’ physical appearance. This fostered acceptance and positivity about their appearance:

*“Even now I find that I can look at myself in the mirror. Whereas before I was pregnant, I’d be like, ‘Okay I’m criticizing myself,’ now I can look at myself and say, ‘You just had a baby. You know give yourself some credit there’.”*

### Maintaining healthy habits

Having developed and maintained healthy habits prior to pregnancy made it easier for women to maintain those habits, preventing excessive weight gain or making it easier to lose weight:

*“I think being healthy prior to pregnancy is important; having those habits and stuff of exercising and eating properly to begin with…so I didn’t have to change too much in my diet…having good habits prior to pregnancy helps with your weight gain during and then also the weight loss after.”*

Several women regretted they had not focused on weight loss prior to becoming pregnant and felt that preparing for future pregnancies would help with their overall health and wellbeing, in addition to appropriate GWG:

*“I think I know that if we are to have a third I really need to go into it the healthiest I can be, because my health will deteriorate…And so I wished I had continued to get in better shape because I know it’s hard on my body and I felt it more this time because I was bigger than I was the last time, I felt more pain in different areas, so I know I need to go into it sort of strong as I can.”*

Challenges to maintaining healthy habits included fatigue, lack of time, support, motivation, unrealistic expectations of nutrition and exercise, family responsibilities, cost, pregnancy-related complications, weather, and not regarding nutrition and exercise as a priority.

One of the biggest factors associated with engaging in healthy behaviours reported by new mothers was the unexpected amount of time required to care for their infant, which many women did without partner or social supports, in addition to their normal day-to-day duties. With so many responsibilities, exercise and healthy eating were often not a priority, and essential tasks such as eating, let alone eating a healthy meal, was often difficult:

*“I think it’s a challenging point in someone’s life because you go through your whole pregnancy being very, very motivated to watch what you’re eating and drinking and all those things and you’re very motivated. I think there’s this misconception that when you’re at home you’re going to have all this time to make healthy meals and you never would have probably thought that you would have five minutes to scarf down, you know, a cucumber or something, right. But I think the challenges of being busy with a baby make your diet and exercise challenges that much more unique.”*

Cost was described as a factor for both food and exercise opportunities. Gym memberships or at-home exercise equipment was expensive and often prohibitive to people with limited income, “*It would be nice if there were more options that were cheap because as soon as it’s not free, I’m not sure that I could afford it.”*

From the perspective of participants, complications and general side effects of pregnancy, such as sore joints and bigger size, made exercise and healthy eating difficult. Some women felt they were not in control of their own bodies. One participant who suffered from extreme nausea recalled, “*I was sick up until the very last moment…So whatever I keep in, I keep in. So it wasn’t always healthy choices, but it stayed down.”*

Although there were challenges to healthy habits, participants also found strategies that aided them in achieving a healthy diet and exercise. Warmer weather and low-cost food and exercise options was perceived as helpful:

*“I think that the time of year really helped with the eating and diet too just because it was summer…we were out hiking every weekend, out for walks three and four times a week and that was easy to do.”*

The women reported that having support from family was crucial for being able to balance newborn care with family responsibilities and their nutritional and exercise goals. Support to watch the baby, or other children, provide meals or help around the house gave them the time to make wellness a priority.

Accountability through apps, or group activities forced people to get out and do something to achieve their goals. Also, understanding the benefits of exercise and nutrition was described as being very helpful for making healthy habits a priority. Once participants understood or had experienced the benefits of healthier behaviours, they suggested it became easier to continue to make their health a priority:

*“I would be so exhausted some days before I went to work, and I’d be like “I don’t know how I’m gonna do this at all”. And then once I started, I felt like a million dollars. I felt so much better…So one thing I learned was that being fit really helped me for energy. It was really necessary; and that fatigue wasn’t always something that I should have listened to.”*

### Questioning gestational weight gain counseling

The majority of participants stated that the weight gain recommendations for pregnancy were not helpful and or realistic to achieve:

*“I think a lot of those goals are kind of bogus and ridiculous…I don’t like to look at the numbers, to pay attention to when doctors are like, “You can only gain 10 pounds,” well it’s not realistic either. You have to be able to survive and get through pregnancy, and if it’s eating a lot of junk food for a little bit and you gain a lot of weight, who cares.”*

A central concern that participants raised was the accuracy of the BMI-based guidelines. Participants described that BMI was not an accurate measure of a person’s health status as it failed to consider muscle mass, activity levels, eating habits and body type. Some commented that in their view, BMI had been ‘debunked’ as not being a good measure of obesity and health. The women suggested that a more personalized guideline, one not focused primarily on weight, was needed. One participant pointed out that particularly for women, weight is often used as a proxy measure for health, which doesn’t address the behaviours that lead to better health outcomes:

*“It says that you’re supposed to gain 25 pounds and you gain 30 or 35, but if you gain 70… when should you start worrying and being concerned about it? And I think that’s what needs to be more focused on instead of like this range, of like, “you are normal, and you are underweight, you are overweight, you are obese.”*

Finally, some participants spoke about how their weight seemed beyond their control, which was frustrating to those who experienced excessive weight gain despite doing everything ‘right’.

There was a notable gap described by women regarding counselling and follow-up from health care providers regarding weight guidelines. Some women didn’t receive any weight recommendations from their health care provider, and many didn’t receive any follow-up throughout their pregnancy or postpartum. Women reported that once they started gaining above the recommended weight, they didn’t know what to do as there was a lack of counselling from their provider. One woman remarked:

*“So when you get to the top [weight recommendation], you’re like, well what do I do now? Because when you’re not pregnant I know what to do, but what do I do now exactly? Do I let it continue to climb? And then how will my health care provider respond to that?... But you just go and get weighed and measured and like call it a day.”*

Overall, participants indicated a lack of understanding of the consequences of excessive GWG for both mother and baby. A common belief shared in the focus groups was that the baby ‘took what it needed’, so that a poor diet or a lack of exercise didn’t impact the baby’s health. The women stated that health care providers did not explain why there are weight gain recommendations or the risks of gaining above or below:

*“I don’t think I had that conversation. There wasn’t, like, if you go above the number these are the risks. I think I knew that from other places but not from my primary health care provider.”*

### Learning through resources

The availability of information was a key issue raised by participants. While women reported using a multitude of resources, such as healthcare providers, the internet, phone apps, prenatal classes, and their peers, they expressed frustration regarding the scarcity of resources that specifically addressed the postpartum period. There were many more resources available for pregnancy-related topics in comparison to postpartum, and most resources for postpartum were focused on mental health:

*“I find there was more resources for health during pregnancy then there is while breastfeeding after pregnancy, as to like, what supplements you can take and what’s safe to have while you’re pregnant, there’s so many resources about that…. but there’s not so much for after and when you’re breastfeeding.”*

According to the women, there was a lack of follow-up from healthcare providers regarding how to lose excess weight in a healthy manner postpartum, and there was little information about what types of exercises, in what duration and intensity were safe and effective:

*“I think if they made it [information] more available for after. Like saying, here’s postpartum what it looks like your body’s doing, you need to give it time to let it recover, but you need to also be mindful of this and this and this.*”

Resources specific to postpartum exercise were described as being limited, including those, which addressed diastasis recti separation, pelvic floor strengthening, and how exercise impacted breastfeeding. Women stated a preference for getting information directly from their health care provider, or public health programs because they didn’t have to question the source, unlike information coming from the internet or apps:

“*You get inundated with a lot of information that you… you really have to look at where it’s coming from because there’s a lot of chat rooms and people’s personal experiences, so I always tried to stick to like, sites that had like a doctor that had written or a dietitian or something.”*

## Discussion

This study explored experiences related to nutrition, exercise and weight gain among women during early postpartum. We found that women in the postpartum period may experience a significantly altered body image, and incongruence between their goals and expectations and their lived experience of initiating and maintaining exercise and healthy eating. This disparity between expectations and experience may be due to the overall paucity of information and lack of importance paid to this time period in women’s lives. According to our participants, postpartum body image is far more complex than simple dissatisfaction with weight. Many postpartum women felt dissatisfaction and guilt over how their body looked and changed after pregnancy. This is consistent with existing literature on the topic, which outlines that, similar to our participants, positive body image fluctuates throughout pregnancy and then decreases in postpartum [50, 64–66]. Similar to our findings, several studies have found that women felt more negatively about their bodies postpartum than they had expected and felt it would have been helpful to be counselled on what to expect for recovery postpartum [50, 67]. While the scientific literature primarily reflects the negative experiences of women’s postpartum body image, there are social movements taking place to encourage positive body image in the postpartum [68, 69]. These movements illustrate and reinforce the common experience of negative postpartum body image and demonstrates a greater need for research in this area.

Our findings highlight the need for establishing healthy habits relating to nutrition and exercise prior to pregnancy. As outlined by our participants, these pre-existing patterns and healthy behaviours were crucial to achieving appropriate GWG and maintaining proper health through pregnancy and postpartum. Overall, pre-pregnancy factors contribute 74% to excessive GWG, in comparison to modifiable pregnancy related and pregnancy-related health conditions which only contribute 11 and 15%, respectively [29]. This means that the most effective time for interventions that limit excessive GWG is before pregnancy. Many sources agree that the optimal management of obesity in childbearing women must include pre-conception counselling, and pre-gravid weight loss programs, in addition to counselling and follow-up during pregnancy [70–72]. The Academy of Nutrition and Dietetics’ position statement suggests that for overweight and obese women behavioural counselling to improve diet and physical activity should start prior to pregnancy [72]. However, our findings suggest that this recommendation should be expanded to include all women of reproductive age. We noted a lack of knowledge among participants regarding any consequences of excessive weight gain in pregnancy. Pre-conception counselling and health teaching during pregnancy should address maternal and fetal risks associated with excessive weight gain, risks of weight retention postpartum, strategies for adopting healthy eating and activity, as well as the benefits of lifestyle modifications and interventions that can combat excess weight gain [71, 72].

The postpartum period brings unique challenges and barriers which historically have been poorly understood. These include lack of time, support and motivation; family responsibilities; cost barriers; breastfeeding concerns and unrealistic expectations [5, 67, 73–77]. In addition, the nuances of the postpartum experience have not been well addressed by health care providers or by traditional sources of information. The results of our study highlight that women may receive limited counselling from care providers specific to the postpartum period, particularly addressing approaches to healthy habits while transitioning into a new or expanded parenting role [27, 78–80]. Evidence based information on this topic is lacking and not easily accessible. For example, clinical practice guidelines addressing postpartum exercise are scarce [79, 81] and those that exist are brief and lack strategies practitioners could use when counselling patients [80]. An additional barrier reported by healthcare providers is a lack of confidence in providing exercise, nutritional or GWG counselling in pregnancy or postpartum, due to a lack of knowledge, skills or resources [82–84]. Future research should involve the development of resources, knowledge translation strategies and counselling tools specific to postpartum.

Our research shed light on participant’s distrust and disconnection with GWG recommendations. They dismissed these recommendations, preferring to use their personal experiences or anecdotal evidence to determine how much weight to gain during pregnancy, rather than adhering to their health care provider’s recommendation or target. This distrust stemmed from a poor understanding of the long-term consequences and relevance of GWG recommendations, and from scepticism concerning the validity of BMI as an accurate measure of health during pregnancy. While this echoes similar research examining patient views of BMI, this is a new insight into the perception of BMI by patients in relation to GWG [85, 86]. This distrust of recommendations needs to be explored in greater depth for future development of counselling and knowledge translation tools about weight gain and retention during childbearing if they are to be successful. The women in our study articulated wanting personalized approaches, on-going follow-up, and an improved understanding of the relevance and consequences of weight gain or retention. In previous research only a minority of women were informed of the risks of weight gain by their providers, women who were overweight or obese were less likely to have been given this information [44, 45, 87] and very few receive on-going, personalized counselling for how to operationalize recommendations [83].

Strengths of our study included our grounded theory approach which enabled the findings to be grounded in the experiences and data provided by the participants. This gave participants the flexibility to guide the discussion to topics they found salient regarding their postpartum experiences and allowed the researchers to refine their data findings through theoretical sampling. Our findings may be transferable to other postpartum populations in urban settings.. However, we recognize that women interested in or concerned about weight control and health during pregnancy may have been more likely to participate in the trial and to attend the focus group. A limitation of our study was the low participation rates in the focus groups, likely due to the demands of new parenthood and modified family roles resulting in participants having limited free time [5]. Additionally, there was a period when staff were unavailable to run the information sessions, therefore the focus groups were postponed. This gap in contact with participants may have negatively impacted retention.

## Conclusion

Our study has highlighted several challenges that women face in achieving healthy behaviours and weight loss in postpartum. Healthy habits and strategies that are responsive to the characteristics of this transitional time in a woman’s life have been neglected in the research, counselling practices, and resources available for both postpartum women and health care providers. We recommend that pre-pregnancy counselling be expanded to all women of reproductive age for effective pregnancy and postpartum weight management.

## Data Availability

The datasets generated and/or analyzed during the current study are not publicly available due lack of consent from the study participants to share the data publicly but are available from the corresponding author on reasonable request.

## Abbreviations

BMI: Body mass index
GWG: Gestational weight gain
RCT: Randomized controlled trial
